# Supplementary figures and images for: Mutation of regulatory phosphorylation sites in PFKFB2 does not affect the anti-fibrotic effect of metformin in the kidney
Source: PLoS One. 2023 Feb 9;18(2):e0280792. doi: 10.1371/journal.pone.0280792 (PMC9910667; doi:10.1371/journal.pone.0280792)

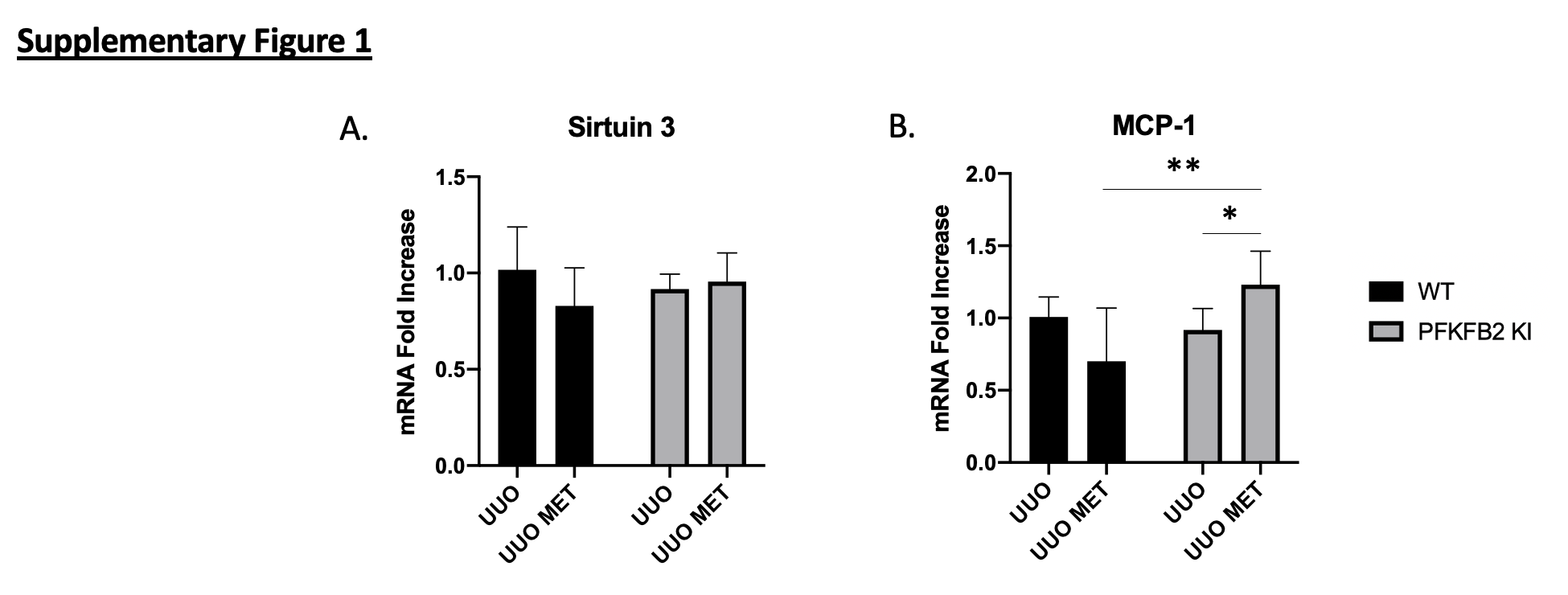

Supplement: S1 Fig — Measurement of mRNA expression via RT-PCR of other markers for WT and PFKFB2 KI UUO ± metformin kidneys (A, B). There was no significant difference in expression of Sirtuin 3 between groups (A). Expression of monocyte chemoattractant protein-1 (MCP-1) was increased in PFKFB2 KI UUO + metformin kidneys compared to PFKFB2 KI UUO controls (B *p = 0.0472) and WT UUO + metformin comparators (B **p = 0.0014). Mean + SD. (TIF) [file pone.0280792.s001.tif]
