# Supplementary material for: Mutation of regulatory phosphorylation sites in PFKFB2 does not affect the anti-fibrotic effect of metformin in the kidney
Source: PLoS One. 2023 Feb 9;18(2):e0280792. doi: 10.1371/journal.pone.0280792 (PMC9910667; doi:10.1371/journal.pone.0280792)
Supplement: S1 Raw images — (PDF) [file pone.0280792.s002.pdf]

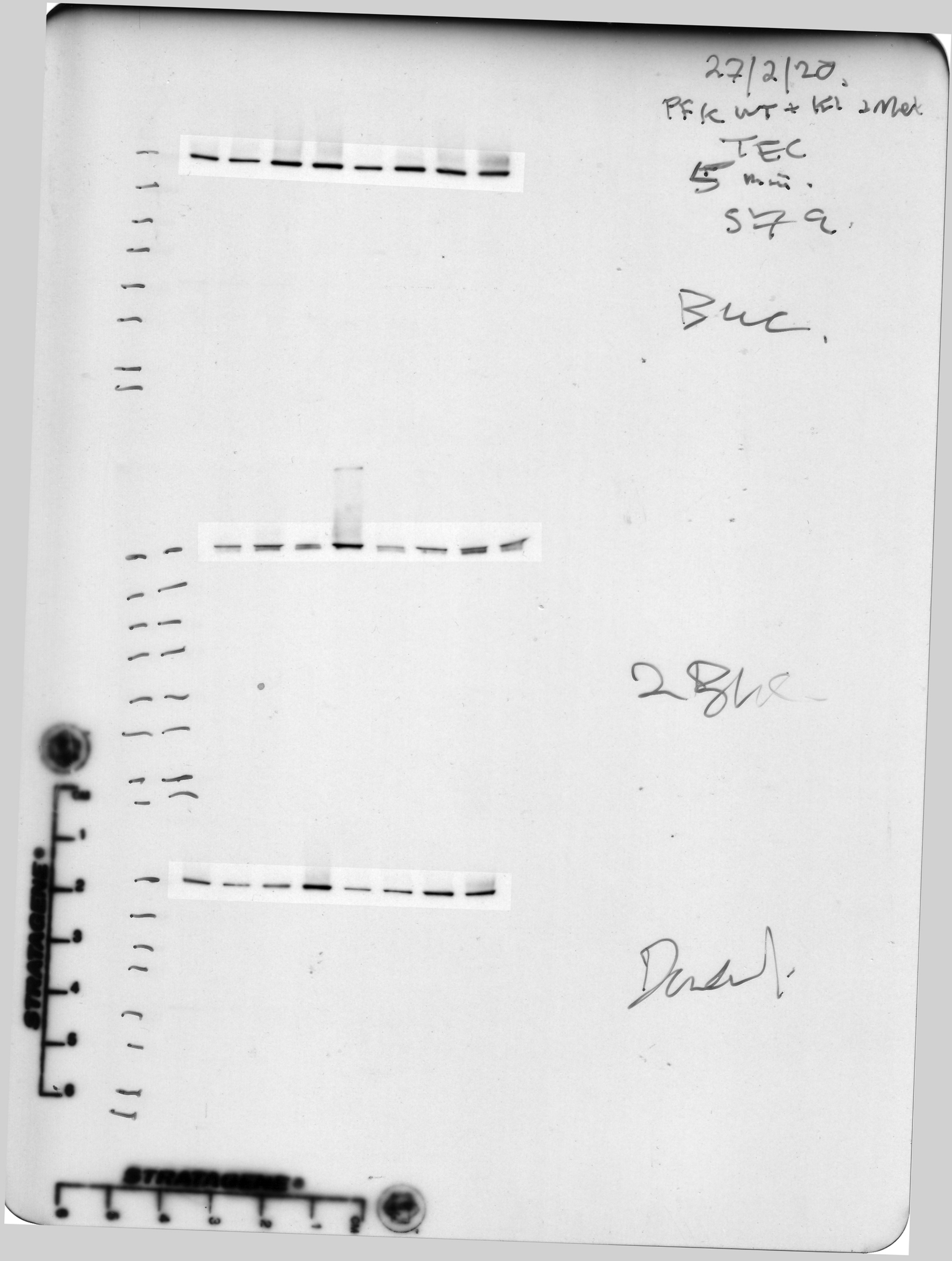
27/2/20.
TEC
5 min.
S79.
STRATAGENE
STRATAGENE

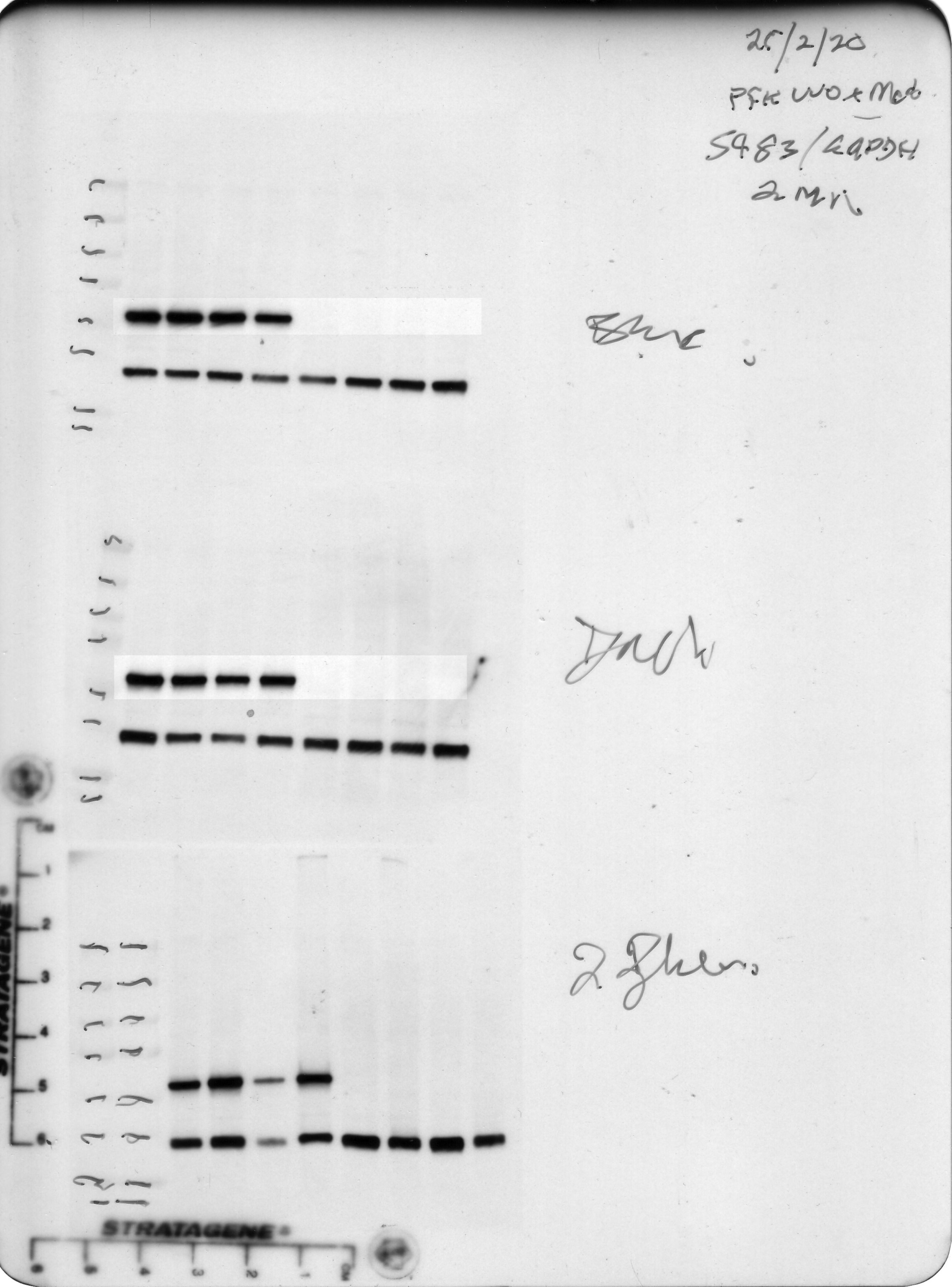
25/2/20
5483/GAPDH
STRATAGENE
STRATAGENE

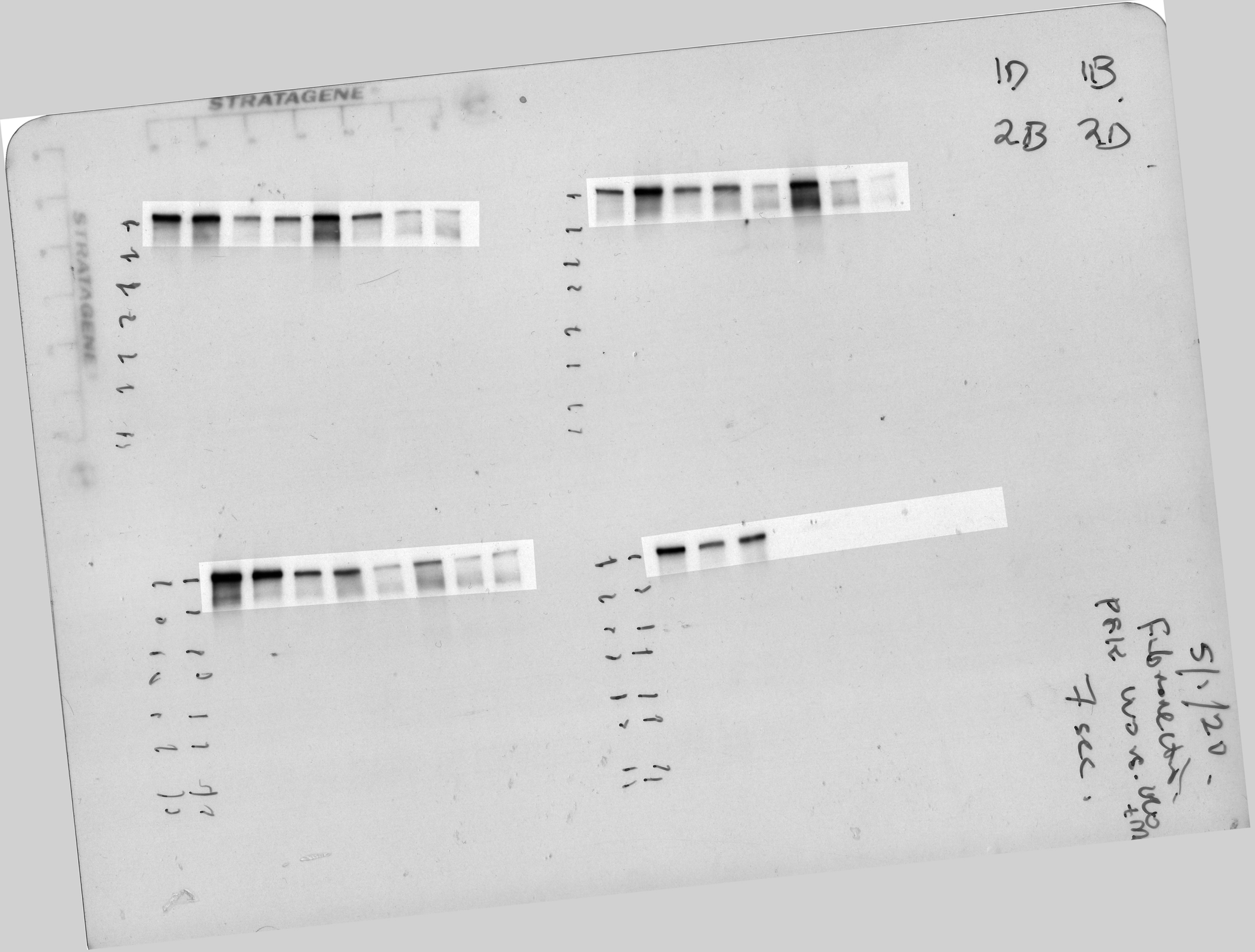
STRATAGENE
1D 1B.
2B 2D
5/1/20 -
7 sec.

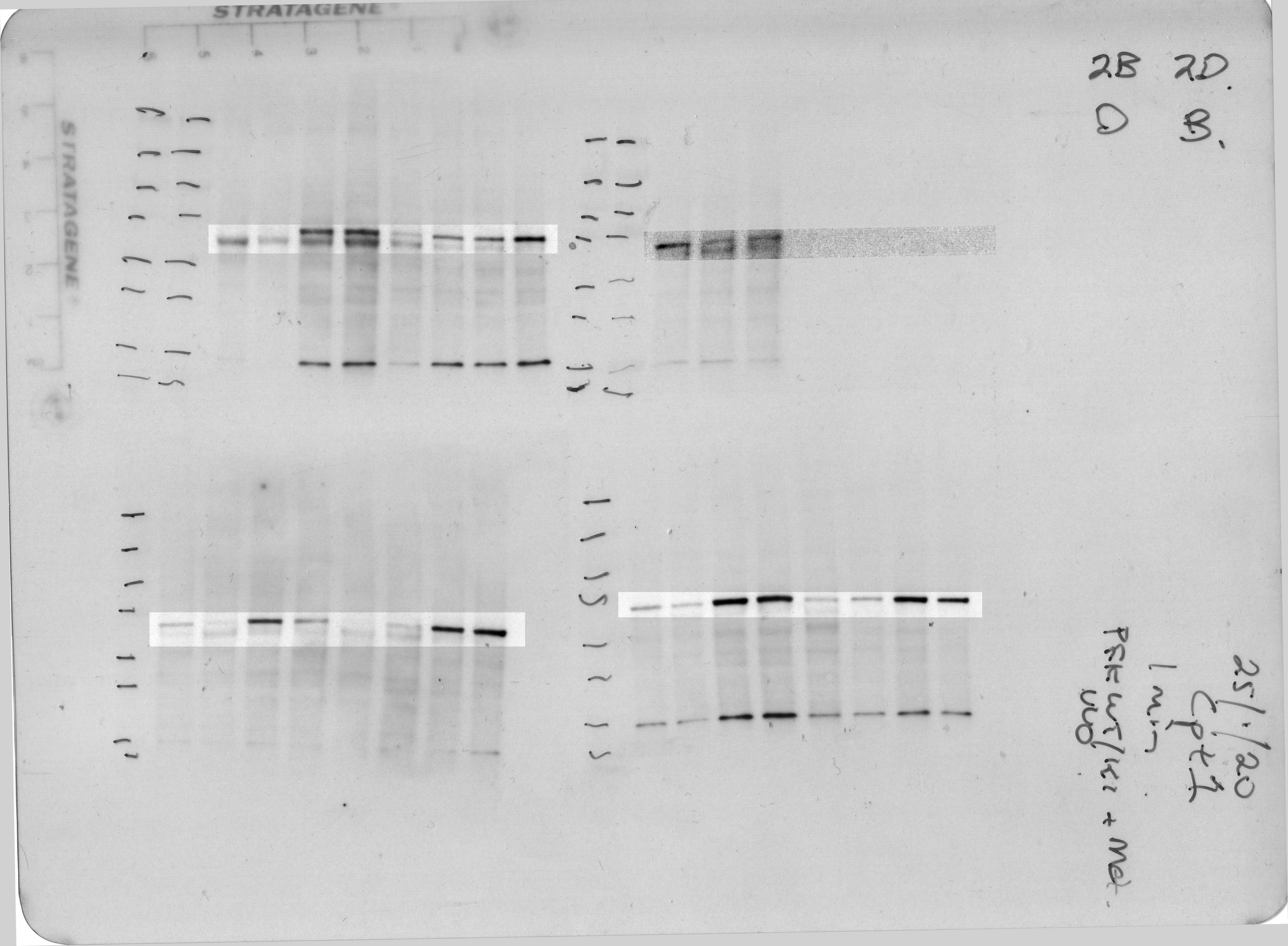
STRATAGENE
2B 2D.
D B.
25/1/20

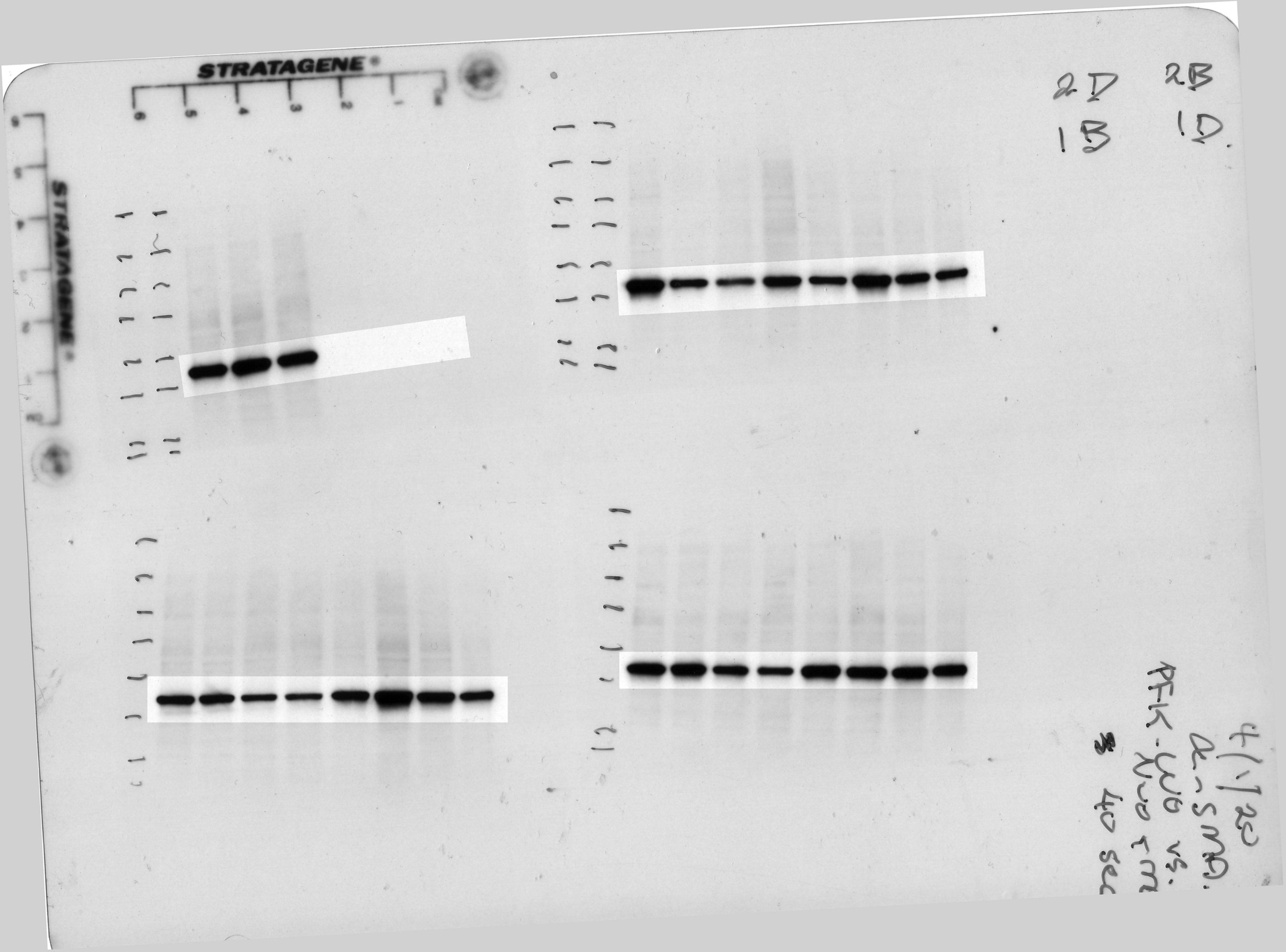
STRATAGENE
STRATAGENE
2D 2B
1B 1D.
4/1/20
α-SMA.
PFK-WO vs.
WO
40 sec

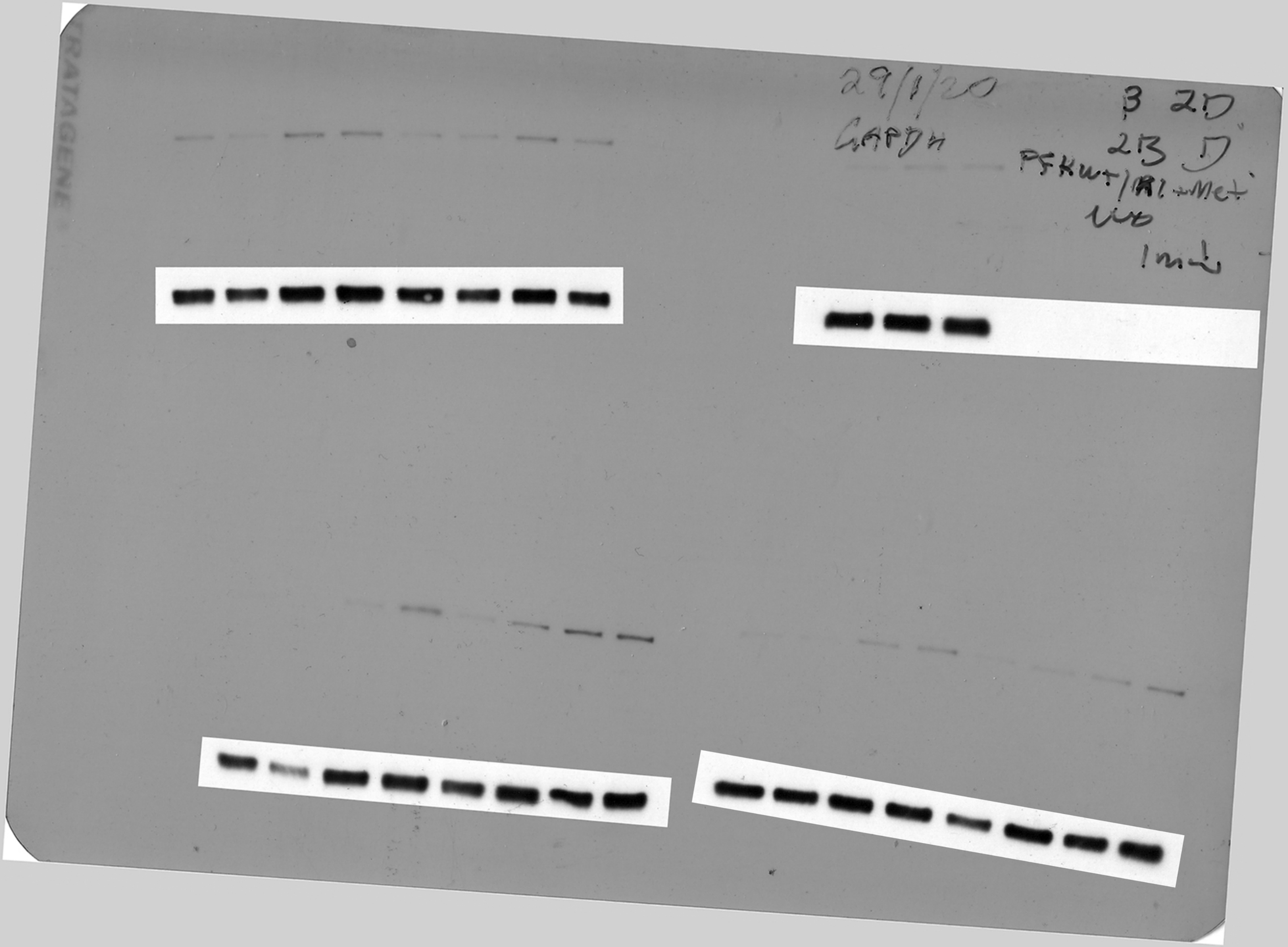
29/1/20
GAPDH

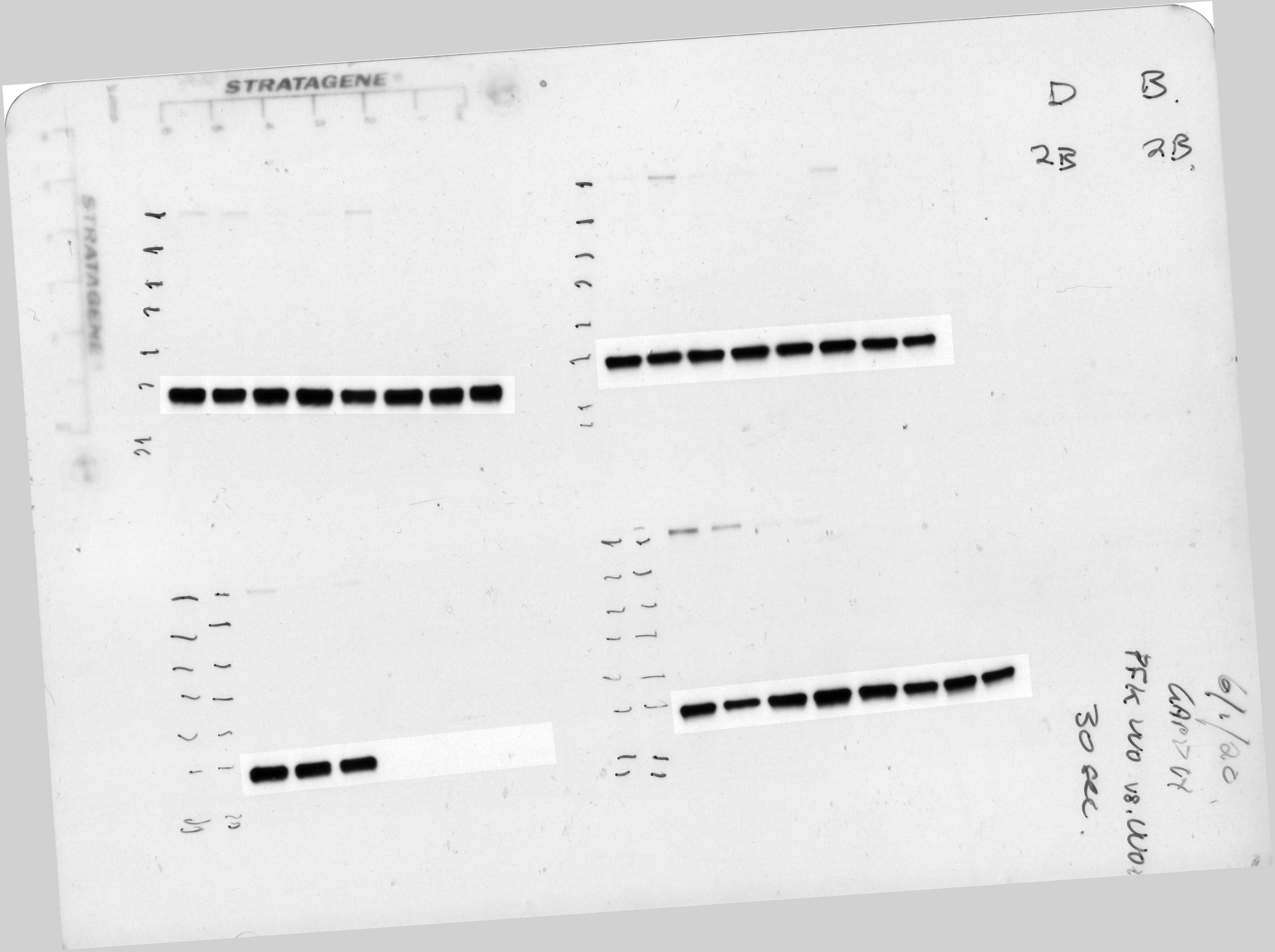
STRATAGENE
D
B.
2B
2B.

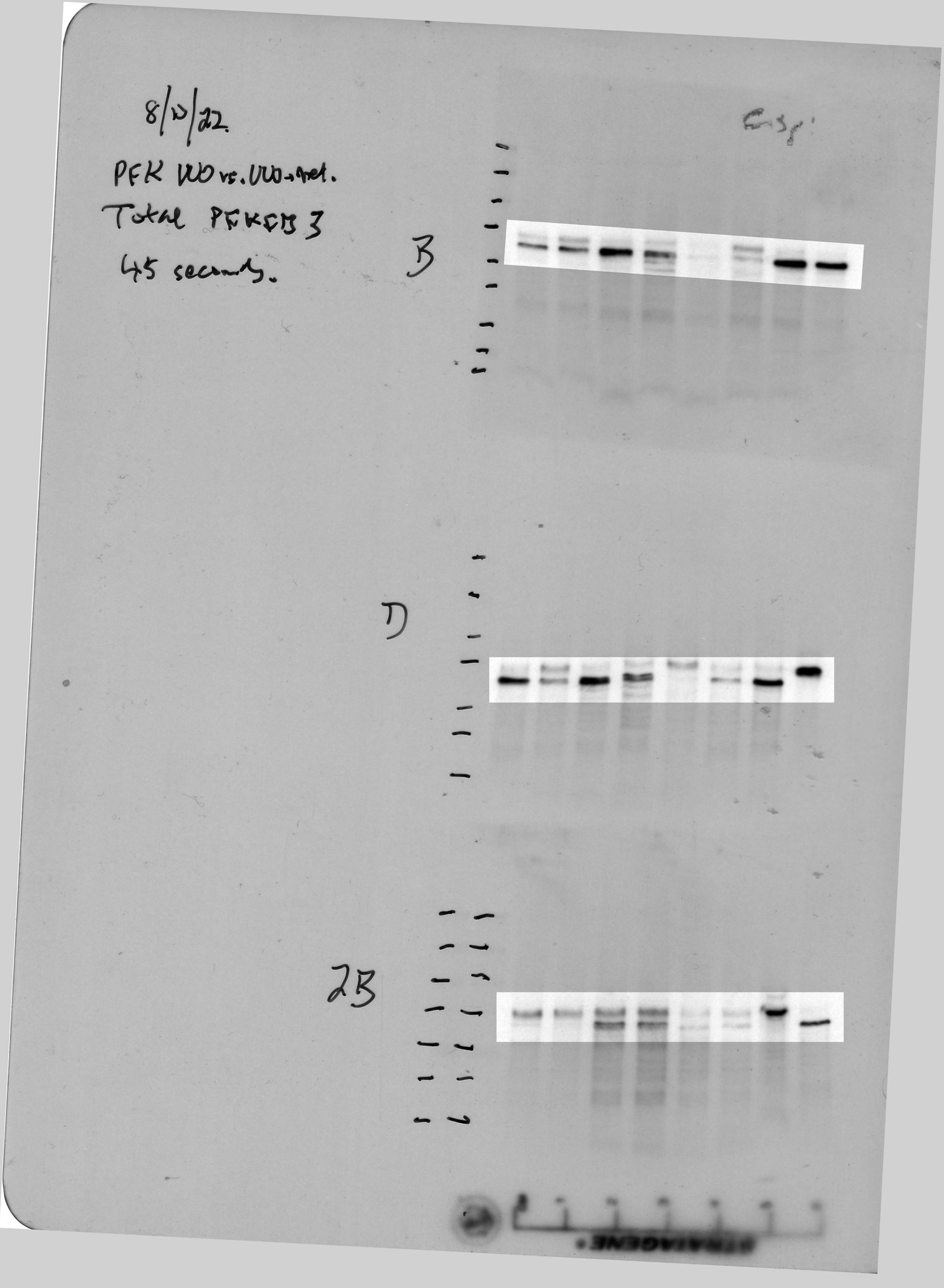
8/10/22.
PFK UUO vs. UUO-met.
Total PFKFB3
45 seconds.
B
D
2B

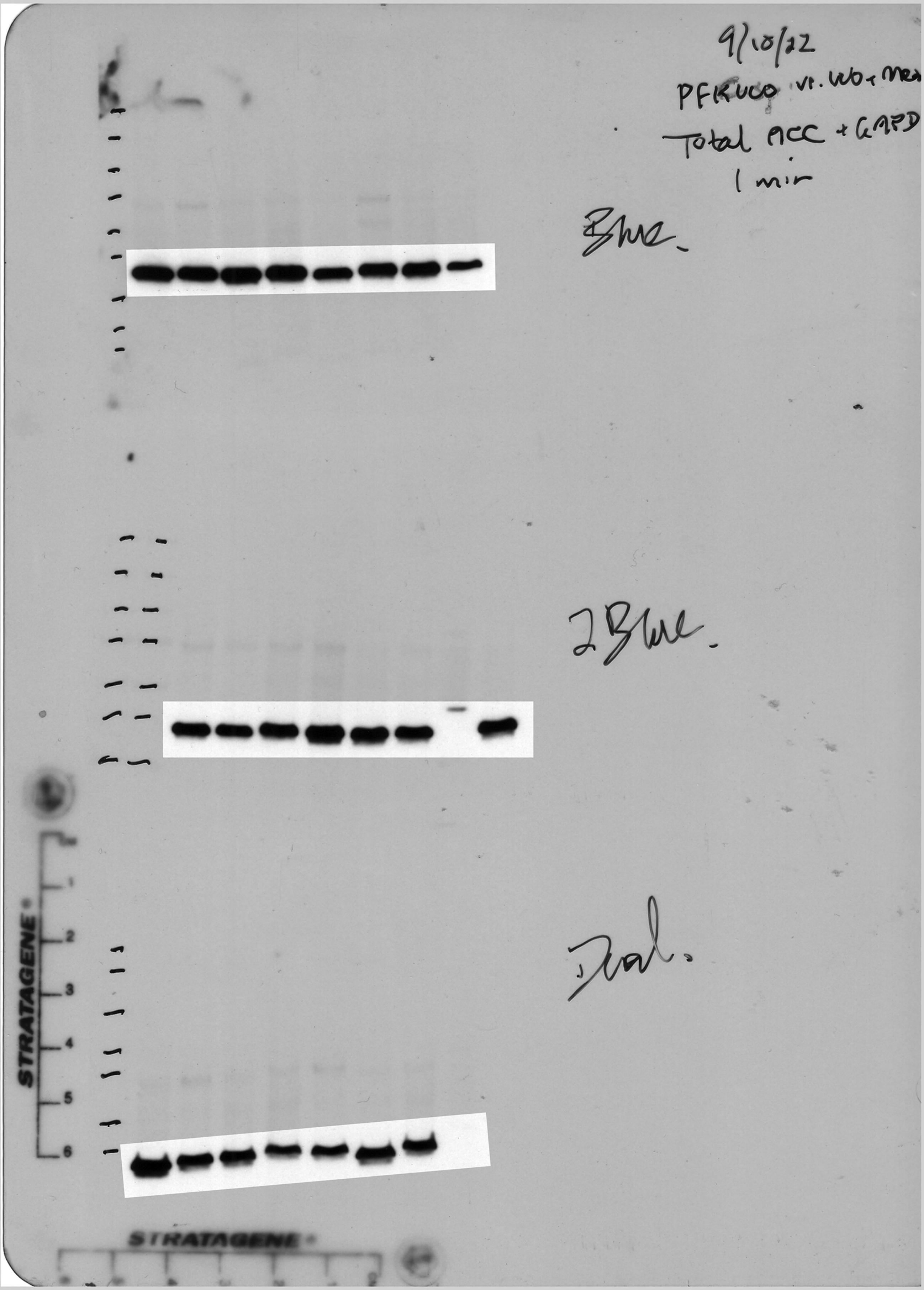

9/10/22
PFKKO vs. WO + Mex
Total ACC + GAPD
1 min
Blue.
2 Blue.
Dual.
STRATAGENE
STRATAGENE

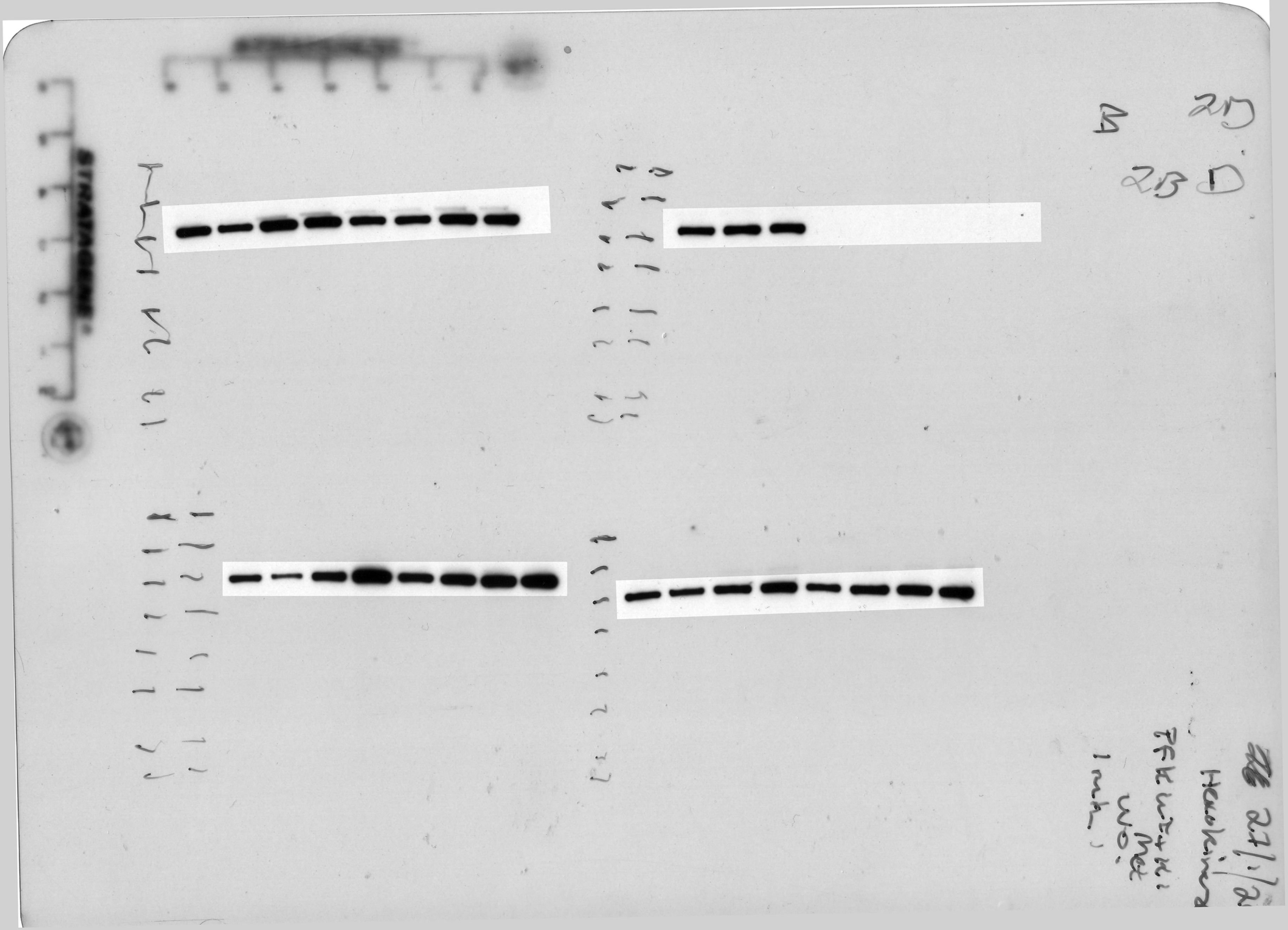

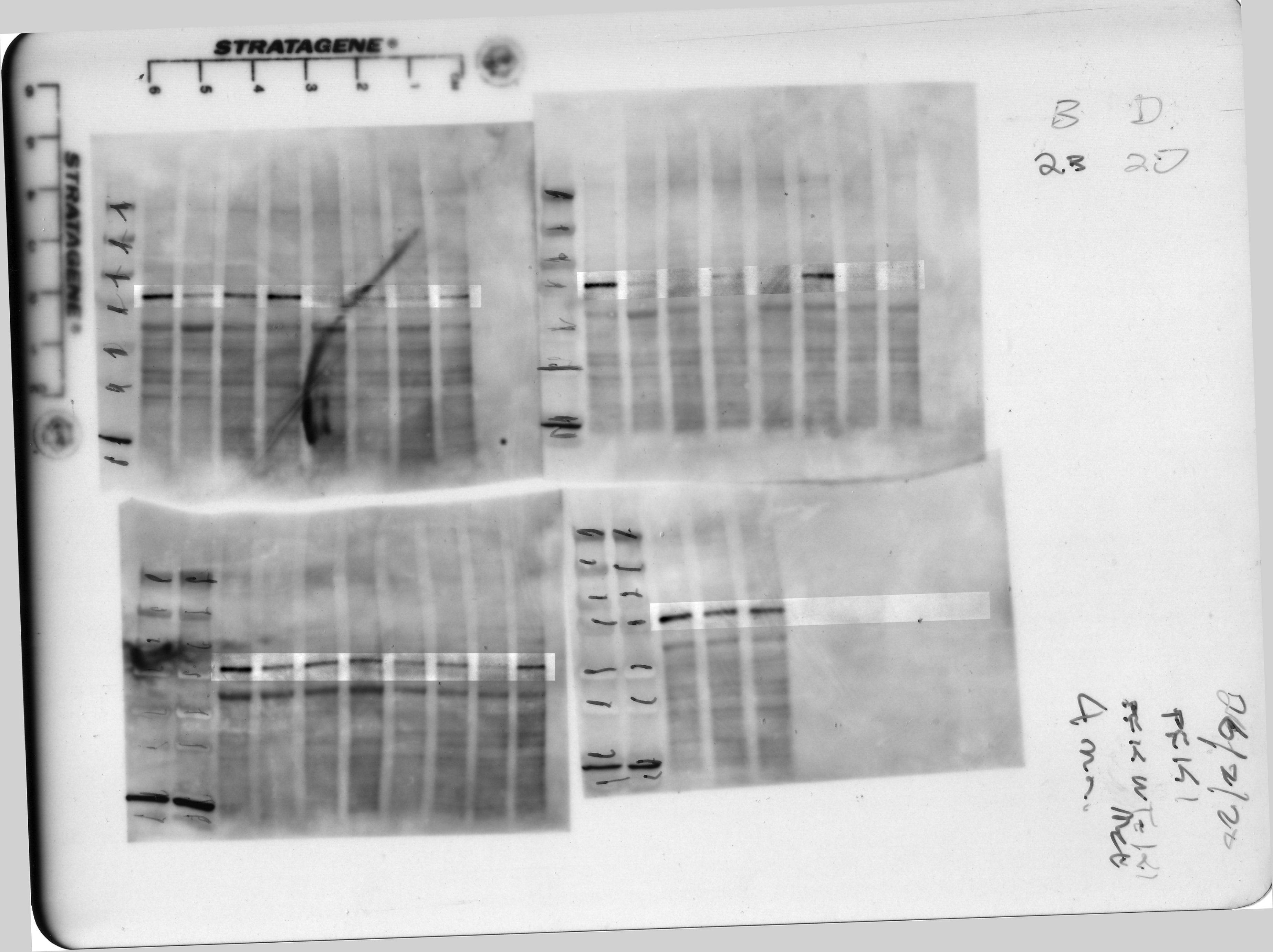
STRATAGENE
B D
2.3 2D

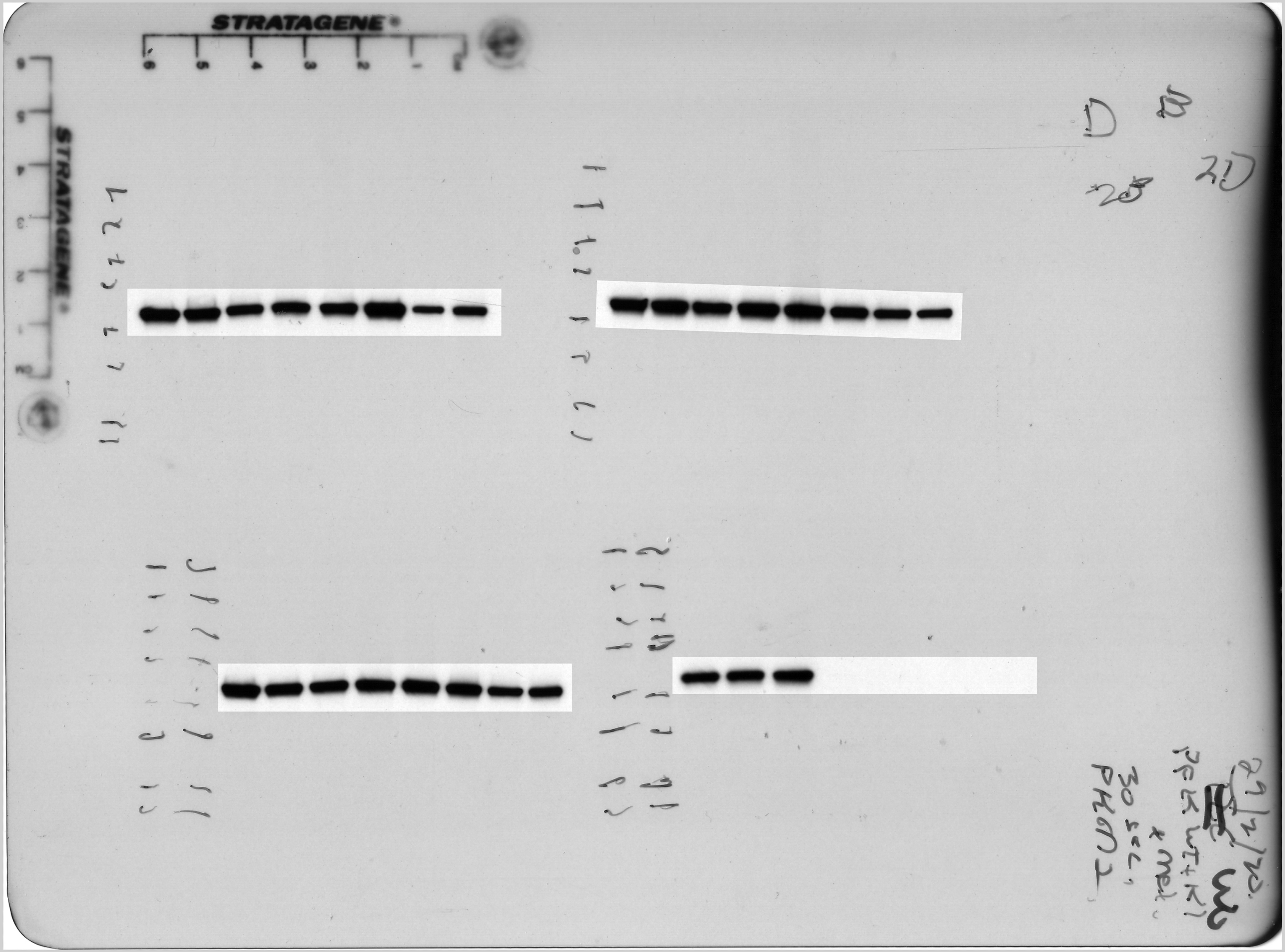
STRATAGENE

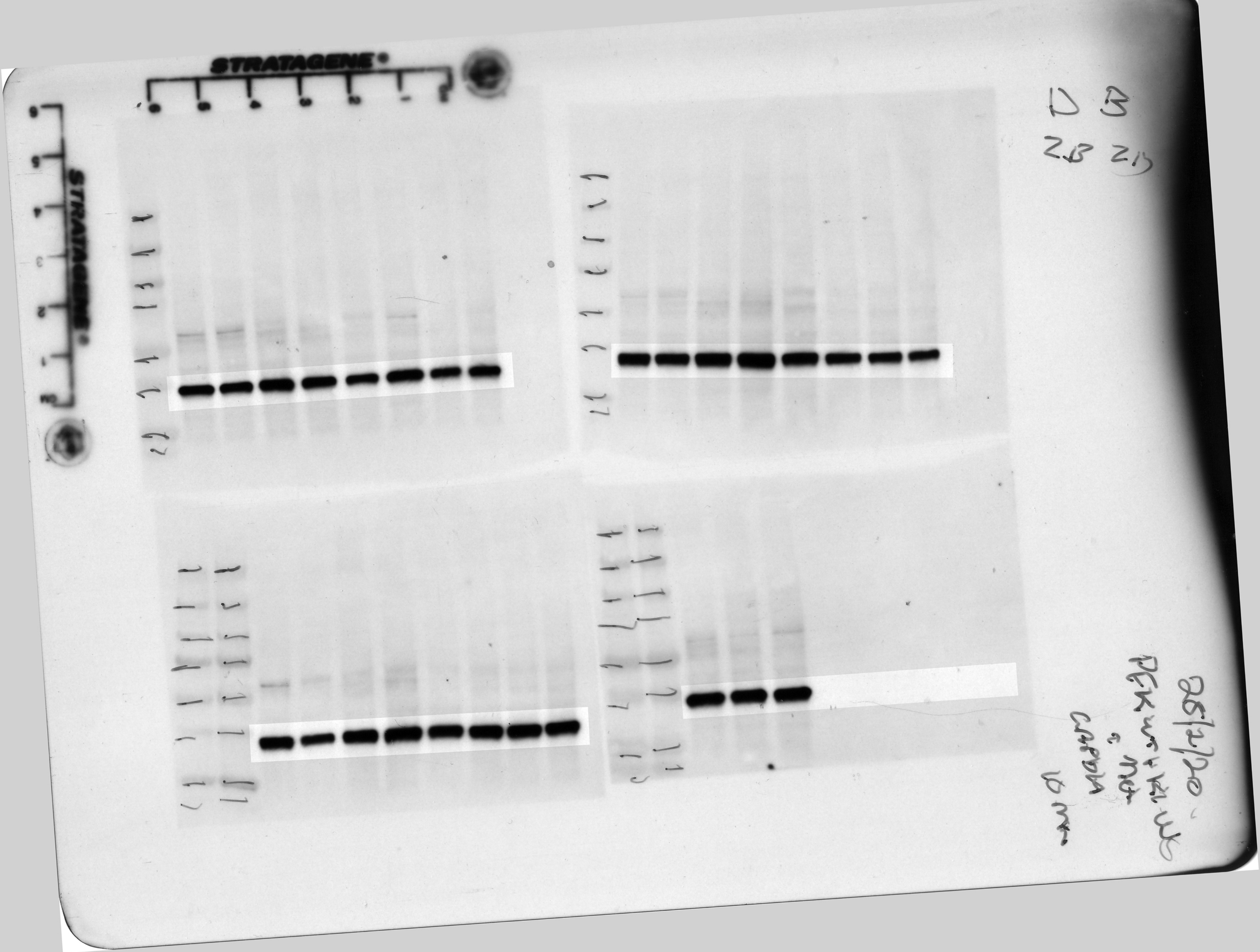
STRATAGENE
STRATAGENE
PEK wt + KL WB
GAPDH
10 min

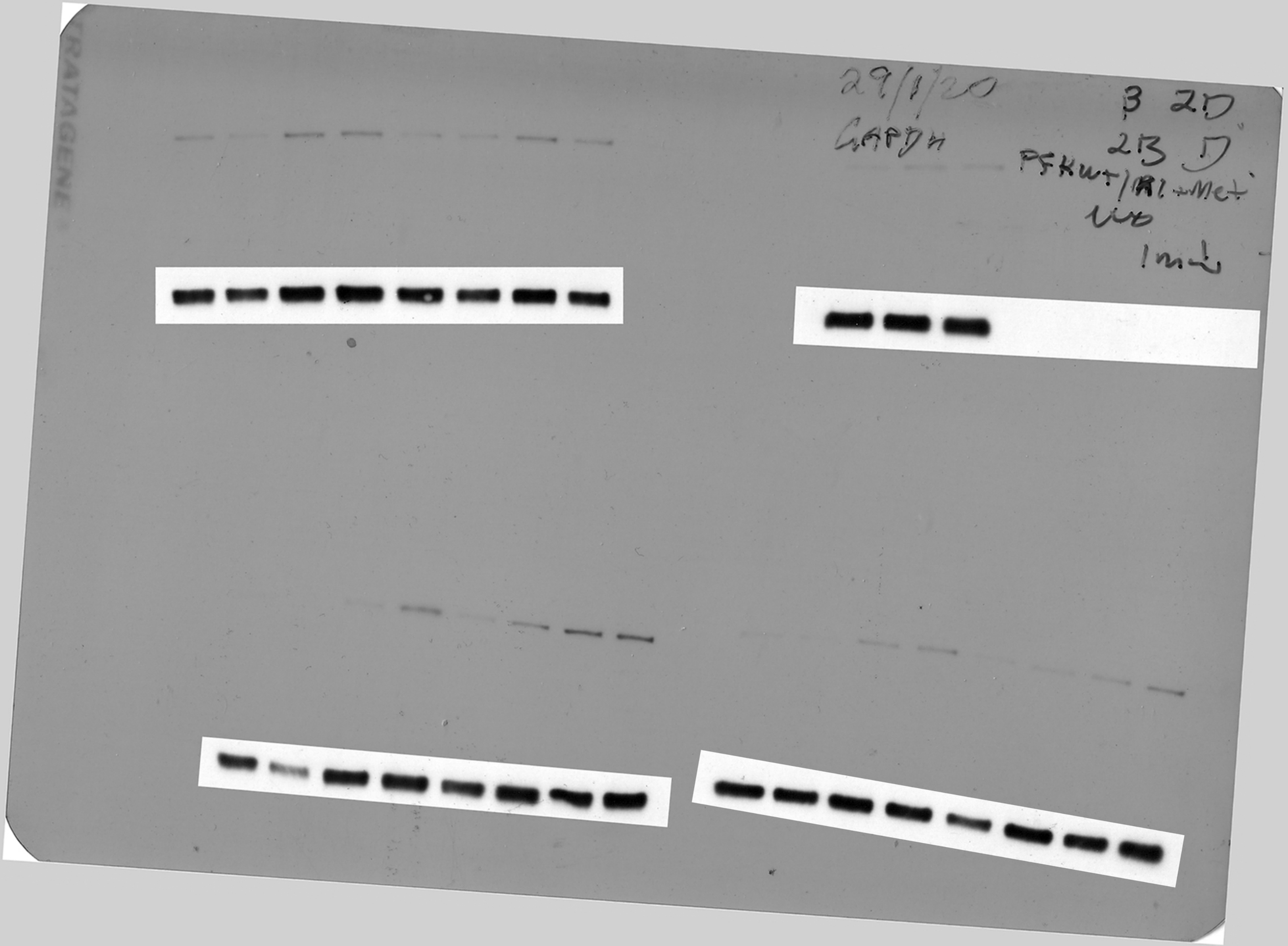
29/1/20
GAPDH
3 2D
2B D
1min
STRATAGENE
