# Supplementary material for: Mutation of regulatory phosphorylation sites in PFKFB2 does not affect the anti-fibrotic effect of metformin in the kidney
Source: PLoS One. 2023 Feb 9;18(2):e0280792. doi: 10.1371/journal.pone.0280792 (PMC9910667; doi:10.1371/journal.pone.0280792)
Supplement: Attachment — Submitted filename: Response to Reviewers.docx [file pone.0280792.s003.docx]

28^th^ April 2022

Dear editors of PLOS ONE,

On behalf of the authors, I would like to thank you for considering our work and the thoughtful observations made. In response, we have undertaken additional histology and RT-PCR work to further confirm the original findings and have substantially modified the figures. The changes have been indicated in the manuscript with tracked changes. Please find below our responses to the comments of the reviewers. We have also added an additional author, Mardiana Lee, who contributed significantly to the further histology work. I have also, as requested, included an amendment of the Funding Statement to comply with the journal’s requirements, which is included at the end.

Regards,

Dr Geoff Harley

Nephrologist

Austin Health Renal Lab

Reviewer #1: In the present manuscript Harley et al. reported the anti-fibrotic effect of metformin in vivo in a mouse model of renal fibrosis induced by unilateral ureteral occlusion (UUO). This anti-fibrotic effect of metformin was independent from the rate of glycolysis. The authors used a 7-day UUO model in both wild-type and genetically modified mice (PFKFB2 KI) lacking a key regulatory point of glycolysis (mutated phosphorylation sites of phosphofructokinase-2) resulting in a reduced ability to increase glycolysis in the kidneys. Despite this change, metformin continued to reduce fibrosis in this UUO model.
The goals are clear, the manuscript is well-written and readable, however, there are some typos that should be corrected in the final version.

Nevertheless, there are major comments need to be addressed:
**1. The authors used assessed the severity of renal fibrosis by assessing some key markers of fibrosis including a-SMA or fibronectin at protein or gene expression levels (Figs. 2-3). However, showing representative micrographs (e.g.: Masson’s trichrome or Sirius red staining of the affected kidneys would be a nice addition and improve the quality of the paper.**

We have undertaken additional histology work to confirm these findings using quantitative analysis of a Masson’s trichrome stain and added this as Figure 3. In brief, it confirmed the anti-fibrotic effects of metformin in both WT and PFKFB2 KI UUO groups, similar to the existing Western blot and RT-PCR data. We have updated the results section and abstract to include this additional data.

**2. Please put in data from sham operated animals shown in a separate, supplementary figure set.**

Our group has previously published data regarding the comparison between sham and UUO-operated mice using an identical UUO technique for both WT and PFKFB2 KI mice (Lee et al., 2020) so this was not repeated as part of this paper. We have now explained this point in the results section of the revised manuscript.

**3. Based on the relevant literature, The mitochondrial deacetylase sirtuin 3 (Sirt3) is involved the stress response activating mitochondrial enzymes involved in fatty acid oxidation, amino acid metabolism, electron transport chain activity, etc. Furthermore, Sirt3 deactivation is a key player in renal fibrosis resulting in epithelial-mesenchymal transition. Did the authors assess the role of Sirt3 in this study? How would a dysregulated glycolysis affect the activity of Sirt3 in the presence or absence of metformin in this UUO model?**

We have measured Sirtuin 3 mRNA expression via RT-PCR and not found it to be significantly different between groups. This data has been added as part of Supplementary Figure 2 and explained in the results section.

**4. Did the authors see any changes in parameters describing kidney function (e.g.: NGAL levels reflecting injury or BUN, glomerular filtration rate, etc.) following metformin treatment of either wild-type or PFKFB2 KI mice?**

These parameters were not specifically measured. Since the UUO model leaves the contralateral kidney left intact, in previous studies, we have not found whole body markers of renal function to be useful outcome measures.

**5. Also, please provide information on the levels of other inflammatory cell markers (e.g.: using Ly6C, Ly6G, CD45 immunostainings or RT-PCR) in the injured kidneys.**

These markers were not specifically examined, however, additional RT-PCR analysis of other inflammatory markers has been added to the manuscript as part of the response to the next query.

**6. Similarly, tissue levels of other (more conventional) cytokines should be also measured (e.g.: TNFa, IL-1b, IL-17, IL-18, IL-33, MCP-1, MIP-1a).**

Additional inflammatory markers have been measured via RT-PCR analysis. These include TNF-alpha, MCP-1 and IL-1. They have been included as part of figure 5 and supplementary figure 2. Similar to the fibrosis markers, the inflammation markers F4.80, TNF-alpha, IL-1 and IL-6 were reduced with metformin and not significantly different between WT and PFKFB2 KI mice. Explanation of these data has been added to the text of the results.

**7. Based on Fig 5, both wild-type and PFKFB2 KI mice represented a reduced expression of pyruvate kinase (PKM2). Therefore, measuring the levels of an upstream metabolite (e.g.:2,3 bis-phosphoglycerate) would determine if glycolysis is downregulated in renal fibrosis.**

As mentioned in our discussion, this decrease in PKM2 expression may represent downstream reduced glycolysis as an unexpected action of metformin in this model, however, further studies would be needed to confirm this finding, particularly given the issue of dimers and tetramers of PKM2 being relevant to its overall activity. Assuming this change in Western blot expression correlated with overall activity, further upstream metabolites weren’t specifically examined but would be of interest to explore this effect. However, the manuscript isn’t seeking to make a definitive conclusion about metformin’s effects on the rate of glycolysis, only to show that despite absence of one of the major regulatory mechanisms in control of glycolysis, metformin continues to have significant anti-fibrotic effects. We have added explanation in the discussion (second last paragraph), that a limitation of the study is that glycolylysis was not directly measured.

**Reviewer #2: In the present study “Mutation of regulatory phosphorylation sites in PFKFB2 does not affect the anti-fibrotic effect of metformin in the kidney” the authors analyzed the effect of PFKB2 on kidney fibrosis and the contribution to the protective function of metformin. Here are some major concerns to be addressed:
1. The authors need to examine the expression level of PFKFB1, 3 and 4, as well as their phosphorylation levels in the kidneys.**

As established by the work of Minchenko et al. (Minchenko et al., 2003) and others, PFKFB 1, 3 and 4 exist at lower expression levels in mouse kidney tissue compared with PFKFB2. This was the reason we selected PFKFB2 as the most prevalent isoform. PFKFB1 is not present in the kidney in detectable amounts. PFKFB3 and 4 are present, but we have not attempted further study in view of our focus on the dominant isoform, PFKFB2. Additional information has been added to the first paragraph of the discussion to better explain this point.

**2. The authors need to show the metabolic features of the PFKFB2 KI mice with and without metformin, including glucose, amino acid and fatty acids metabolism.**

As stated in the manuscript, in the methods section under the “Generation of PFKFB2 KI mice” heading, we have previously published the observed phenotype of the PFKFB2 KI mice (Lee et al., 2020). There was no difference in plasma glucose or mouse weight between PFKFB2 KI mice and controls, however, the PFKFB2 KI kidneys were smaller and plasma urea was significantly less. Furthermore, cultured tubular epithelial cells from PFKFB2 KI mice have impaired glycolysis when analysed on the Seahorse analyser (Lee et al., 2020). We have added this information to this section of the methods.

In this revised manuscript we have added Western blot expression of Cpt-1 (Fig 2D) as a marker of fatty acid metabolism, which interestingly was improved with metformin in WT, and followed a similar trend in the PFKFB2 KI mice. This additional data is not explained in the Results section under the “Renal fibrosis in WT and PFKFB2 KI mice” subheading.

**3. A morphological study should be performed on the kidney samples for fibrosis, such as Masson Trichrome staining or Sirius Rid Staining. A hydroxyproline quantification should also be performed.**

Please see our response for query 1 by Reviewer #1. Masson’s trichrome staining was performed to quantify the degree of fibrosis and an additional figure has been added to our manuscript (Fig 3). These data are presented in the Results section under the “Effects of metformin in WT and PFKFB2 KI mice” subheading. Hydroxyproline quantification was not performed given these results were consistent with the pattern of Western blot and RT-PCR data presented.

**4. The authors should show the baseline level of all the parameters in this study by examining and exhibiting the unchallenged WT and PFKFB2 KI mice.**

As discussed above, we have previously published on the phenotype of sham-operated WT and PFKFB2 KI mice using an identical UUO experimental setup (Lee et al., 2020) so that work was not replicated in this paper.

**5. AMPK phosphorylation should be examined in this study.**

This has been added as part of Supplementary Figure 1. We observed that expression of phosphorylated Thr172, the phosphorylation site on AMPK, was increased in PFKFB2 KI UUO kidneys compared to WT counterparts, a finding of uncertain significance. This additional data has been outlined in the Results section, in the second paragraph of the “Renal fibrosis in WT and PFKFB2 KI mice” section.

**6. To define a negative result, the authors need to also establish a positive control in the same condition. Because the authors’ previous study to determine the function of AMPK-ACC pathway in metformin action on kidney fibrosis is performed on a different kidney fibrosis model, it cannot be directly used as a positive control. The authors need to use the ACC KI mice in the current model to establish a positive control.**

Our group has used a similar experimental setup in a previous published work in JASN (Lee et al., 2018). In this work the same experimental setup was used – three days of metformin in the drinking water of the mice then a folate nephropathy model and assessment of the degree of kidney fibrosis seven days later. This was done in both WT and ACC1/2 KI mice as a positive control of the anti-fibrotic effects of metformin. Likewise, in this current study, we demonstrated increased ACC-Ser^79^ activation with metformin even in PFKFB2 KI mice, suggesting ongoing effects on the ACC pathway and leading to our hypothesized explanation of the ongoing protective effects of metformin.

The protective effects of metformin in UUO models of fibrosis have been well established in the past; for instance Cavaglieri et al. used a UUO model in WT C57Bl/6 mice with metformin given one day prior to surgery and the outcome assessed seven days later compared to sham controls (Cavaglieri et al., 2015). They demonstrated a significant protective of metformin based on Western blot and histological measures of fibrosis. The paper by Shen et al. is another example of this (Shen et al., 2016).

**7. Blood creatinine and BUN should be tested**

These parameters were not specifically measured. Since this is a UUO model with the contralateral kidney left intact, whole body markers of renal function are not useful outcome measures.

**8. Why would metformin not increase the phosphorylation of PFKFB2 in wild type mice?**

As discussed in the first paragraph of the results section, PFKFB2-Ser^483^ is not a phosphosite for AMPK, so metformin should have little effect on its phosphorylation. PFKFB2-Ser^466^ is the active phosphorylation site for AMPK, however, due to sequence homology it cannot be distinguished from a similar site in PFKFB3. Hence, demonstration of increased Western blot expression would be unable to distinguish between increased phosphorylation on PFKFB2 or PFKFB3 so would not be valid.

Amended funding statement

G. H. was supported by a postgraduate scholarship from the University of Melbourne. D.P. received a National Health and Medical Research Council (NHMRC) grant 
[www.nhmrc.gov.au](https://urldefense.com/v3/__http:/www.nhmrc.gov.au__;!!Ek5y5249UU0kCz0!jDzNtLWcuQnKjq8sNp7aiJZCGTPhFp2aO0wRcNI_3fE7rIMYI_H7AoZEZ3lwKWhYVJPaVq9SN247IvoW8CtnjA$). There was no additional external funding received for this study.

References:

Cavaglieri, R., Day, R., Feliers, D., & Abboud, H. (2015). Metformin prevents renal interstitial fibrosis in mice with unilateral ureteral obstruction. *Molecular and Cellular Endocrinology, 412*, 116-122.

Lee, M., Harley, G., Katerelos, M., Gleich, K., Sullivan, M., Laskowski, A., Coughlan, M., Fraser, S., Mount, P., & Power, D. (2020). Mutation of regulatory phosphorylation sites in PFKFB2 worsens renal fibrosis. *Scientific Reports, 10*, 14531.

Lee, M., Katerelos, M., Gleich, K., Galic, S., Kemp, B., Mount, P., & Power, D. (2018). Phosphorylation of Acetyl-CoA Carboxylase by AMPK Reduces Renal Fibrosis and Is Essential for the Anti-Fibrotic Effect of Metformin. *JASN, 29*(9), 2326-2336.

Minchenko, O., Opentanova, I., & Caro, J. (2003). Hypoxic regulation of the 6-phosphofructo-2-kinase/fructose-2,6-bisphosphatase gene family (PFKFB-1-4) expression in vivo. *FEBS Letters, 554*(3), 264-270.

Shen, Y., Miao, N., Xu, J., Gan, X., Xu, D., Zhou, L., Xue, H., Zhang, W., & Lu, L. (2016). Metformin Prevents Renal Fibrosis in Mice with Unilateral Ureteral Obstruction and Inhibits Ang II-Induced ECM Production in Renal Fibroblasts. *Int. J. Mol. Sci, 17*, 146.
