# Supplementary material for: Mutation of regulatory phosphorylation sites in PFKFB2 does not affect the anti-fibrotic effect of metformin in the kidney
Source: PLoS One. 2023 Feb 9;18(2):e0280792. doi: 10.1371/journal.pone.0280792 (PMC9910667; doi:10.1371/journal.pone.0280792)
Supplement: Attachment — Submitted filename: PLOS one response reply to reviewers.docx [file pone.0280792.s004.docx]

Dear PLOS one editors,

3^rd^ December 2022

Re: Manuscript PONE-D-21-37485R1

Mutation of regulatory phosphorylation sites in PFKFB2 does not affect the anti-fibrotic effect of metformin in the kidney

Thank you for re-considering our work. The alterations have been indicated in the manuscript with tracked changes. Please find below our responses to the comments of the reviewer.

Regards,

Dr Geoff Harley

Nephrologist

Austin Health Renal Lab

**1. The authors need to show the expression and phosphorylation of PFKFB1, 3 and 4. The present research shows a negative result, attempting to prove that the PFKFB2 function is not important. However, very possibly the other family members have increased their expression and function in compensation. In such case, the conclusion should be rewritten.**

Thank you for this suggestion. In response to this we have undertaken quantification of total PFKFB3 levels by Western blot which, interestingly, showed up-regulation of PFKFB3 expression with metformin – a previously undescribed finding (see new Fig 6). The abstract has been revised to include this observation (see page 2, line 27-28). A description of the observation about increased PFKFB3 has been added to the results section (see page 11, line 281-282). Furthermore, an additional paragraph has been added to the discussion to address this important finding (see pages 13-14). The original figure 6 has been re-ordered as figure 7 to maintain linearity in the manuscript. As outlined in the discussion – PFKFB1 is not present in the kidney in detectable amounts. Likewise, PFKFB4 is present in low levels in the kidney but is not regulated by AMPK, so is unlikely to contribute to the action of metformin (see Minchenko et al. ref 6 of the manuscript). Regarding the interesting point raised here by the reviewer, we have concluded this additional paragraph with an explanation stating that “It is possible that the observed increase in PFKFB3 might be a contributing reason to the lack of effect of mutation of the PFKFB2 phosphosites on the protective action of metformin observed in this study, although this speculation is unproven at the present time”. Finally, we have added three new references to the manuscript (see references 15-17), to assist with placing this additional data about PFKFB3 in context.

**2. In Figure 3, the images show much lower fibrosis in PFKFB2 KI UUO mice than WT UUO mice. It is the mildest in fibrosis in all groups. This is inconsistent with the statistical analysis. The authors should better analyze the data and make a more solid conclusion.**

Thank you to the reviewer for this useful observation. The quantitative analysis in Fig 3 is based on measurement of a set of images that provide coverage of the whole kidney cortex. The methods section has been revised to make our methodology clearer for the reader (see page 6, line 139-141). Furthermore, the legend for figure 3 has been revised to make this point clearer (see page 10, line 239-240). The reviewer has correctly identified that in the previously submitted version of the manuscript that selected representative images did not well represent our quantification based on the whole kidney cortex. To address this, we have revised figure 3, by selecting images that more accurately represent our overall quantified result (see revised Fig 3).

**3. Because the Masson’s Trichrome stain result shown in Figure 3, the authors need to perform hydroxyproline analysis.**

Thank you for this suggestion. We have reviewed the method for hydroxyproline analysis as well as commercial kits that are available. This analysis requires untreated frozen tissue. Unfortunately, the unilateral ureteric obstruction model yields a limited amount of tissue which we have already processed for Western Blot, histology and RT-PCR analyses. There is none left for another analysis. However, we do note that we have already quantified fibrosis by two other methods in addition to the Masson Trichrome histology, namely Western blot for fibronectin and α-SMA (see Fig 2) and RT-PCR for fibronectin, α -SMA, collagen 1 and collagen 3 (Fig 4), and the results were all consistent. Our assessment, therefore, is that our study already contains adequate quantification of kidney fibrosis. If we had capacity to add hydroxyproline analysis to our manuscript, this could provide some further confirmation, but our assessment is that it is very unlikely to alter the overall conclusion of our study. To clarify this point clearly for the readers we have added a new sentence in the results section (page 9, line 216-219) explaining that “Taken together, we note that our observations using a variety of methods, including Masson Trichrome histology, Western blot (fibronectin and α-SMA), and RT-PCR (fibronectin, α-SMA, collagen 1 and 3), indicate that metformin protects against fibrosis in the UUO model, and that this effect is not altered in the PFKFB2 KI mice”.

**4. In supplemental figure 1, why would not metformin increase the AMPK phosphorylation in any groups? If the authors have trouble with AMPK analysis, this data should not be shown in the study, but discussed. However, at least 2 downstream phosphorylation of AMPK should be presented. The authors already showed ACC. Another downstream of AMPK should be shown. PFKFB family members should suffice.**

Thank you for this observation. We agree with the reviewer that it is unusual that AMPK phosphorylation does not increase with metformin. We suspect this may be a methodological issue, as it is well described that AMPK phosphorylation can be influenced by the method of tissue harvesting, and we note that we did not use the freeze-clamp *in situ* method in this study, which is generally regarded as the gold standard for analysis of AMPK activity in tissue samples. As the reviewer has recommended, because of the uncertainty about the validity of this analysis, we have omitted the AMPK blots from the revised version. We have added explanation of this limitation of our study to the discussion (lines 362-364), where we explain that “A limitation of our study is that we do not have data on AMPK activity in the kidneys of the metformin treated mice, which was not possible to analyse as we did not employ a freeze-clamp methodology for harvesting the kidney tissue”.

Another consideration is that the sequence homology between PFKFB2 and PFKFB3 precludes the ability to quantify their phosphorylated forms in isolation to each other. The PFKFB1 and PFKFB4 isoforms are not significantly expressed in the kidney and not known to be phosphorylated by AMPK.

**5. The authors need to use the total protein blotting instead of GAPDH to calculate the phosphorylation level, such as PFKFBs, ACC and AMPK.**

Thank you for identifying this issue. As the reviewer has identified, in Fig 1, we corrected for GAPDH instead of the total ACC and PFKFB2. We acknowledge that there is potential inaccuracy to describe this as quantification of phosphorylation (defined as phosphor/total). As the reviewer has identified, what we have measured is the overall expression of phosphorylated protein (phosphor/GAPDH), which could be influenced by both the phosphorylation state and the total abundance of the protein. Unfortunately, we do not have left over samples to repeat the analysis shown in figure 1. To address this concern, we have substantially revised the wording of the results describing the TEC data in figure 1 (both text and figure legend, see changes to pages 7-8). Specifically, we have explained to the reader that “We note that in this analysis, phosphorylated ACC and PFKFB2 are corrected for GAPDH rather than total ACC and PFKFB2, therefore, there is uncertainty as to whether the changes observed here are entirely explained by a change in the relative phosphorylation state, or whether there is also a contribution from a change in overall ACC or PFKFB2 expression” (lines 180-184).

Regarding the AMPK blot (previous Supp Fig 1), as per point 4 above, we have removed this data from the revised version.
